# Sex and Gender Differences in Ischemic Heart Disease: Endocrine Vascular Disease Approach (EVA) Study Design

**DOI:** 10.1007/s12265-018-9846-5

**Published:** 2018-12-03

**Authors:** Valeria Raparelli, Marco Proietti, Andrea Lenzi, Stefania Basili, On behalf of EVA Collaborators, Claudio Tiberti, Federica Panimolle, Andrea Isidori, Elisa Giannetta, Laura Napoleone, Marta Novo, Silvia Quattrino, Simona Ceccarelli, Eleni Anastasiadou, Cinzia Marchese, Enrico Mangieri, Gaetano Tanzilli, Nicola Viceconte, Francesco Barillà, Carlo Gaudio, Evaristo Ettorre, Giulio Francesco Romiti, Filippo Toriello, Eleonora Ruscio, Tommaso Todisco, Nicolò Sperduti, Giuseppe Santangelo, Giacomo Visioli, Marco Vano, Marco Borgi, Ludovica Maria Antonini, Silvia Robuffo, Claudia Tucci, Maria Virginia Savoia, Agostino Rossoni, Valeria Spugnardi, Annarita Vernile, Mariateresa Santoliquido, Verdiana Santori, Giulia Tosti, Fabrizio Recchia, Francesco Morricone, Roberto Scacciavillani, Alice Lipari, Andrea Zito, Floriana Testa, Giulia Ricci, Ilaria Vellucci, Marianna Vincenti, Silvia Pietropaolo, Daria Amoroso, Lucia Stefanini, Giovanni Talerico, Pasquale Pignatelli, Simona Bartimoccia, Roberto Cangemi, Salvatore Minisola, Sebastiano Filetti, Cristina Nocella, Louise Pilote, Tabeth Tsitsi Jiri, Muhammad Ahmer Wali, Amanpreet Kaur, Anna Rita Vestri, Adriana Servello, Patrizia Ferroni, Clara Crescioli, Cristina Antinozzi, Francesca Serena Pignataro, Tiziana Bellini, Alessandro Trentini, Roberto Carnevale, Carlo Catalano, Iacopo Carbone, Nicola Galea, Giuliano Bertazzoni, Marianna Suppa, Antonello Rosa, Maria Gabriella Scarpellini, Alessandro Coppola, Giulio Illuminati, Paola Mariani, Fabrizio Neri, Paolo Salis, Antonio Segatori, Laurent Tellini, Gianluca Costabile

**Affiliations:** 1 Department of Experimental Medicine, Sapienza University of Rome, Rome, Italy; 2 Centre for Outcomes Research and Evaluation, McGill University Health Centre Research Institute, Montreal, QC Canada; 3 Department of Internal Medicine and Medical Specialties, Sapienza University of Rome, Rome, Italy

**Keywords:** Sex, Gender, Coronary microvascular dysfunction, Sex steroid hormones, Platelet function, Clinical outcomes, Myocardial blush grade

## Abstract

Improvements in ischemic heart disease (IHD) management have been unbalanced between sexes, with coronary microvascular dysfunction considered the likely underlying reason. The Endocrine Vascular disease Approach (EVA) is an observational study (Clinicaltrial.gov NCT02737982) aiming to assess sex and gender interactions between coronary circulation, sexual hormones, and platelet function. Consecutive patients with IHD undergoing coronary angiography will be recruited: (1) to assess sex and gender differences in angiographic reperfusion indexes; (2) to evaluate the effects of estrogen/androgen on sex-related differences in myocardial ischemia; (3) to investigate the platelet biology differences between men and women with IHD; (4) to verify sex- and gender-driven interplay between response to percutaneous coronary intervention, platelets, sex hormones, and myocardial damage at baseline and its impact on 12-month outcomes. The integration of sex and gender in this translational project on IHD will contribute to the identification of new targets for further innovative clinical interventions.

## Rationale

### Integration of Sex- and Gender-Based Approaches in Cardiovascular Research: The Case of Ischemic Heart Disease

Even though the individual’s sex is among the most important modulators of cardiovascular disease (CVD) risk and response to treatment, the integration of sex in research and clinical decision-making is yet overlooked [1–3]. In the era of precision medicine, reporting on sex- and gender-related variables in clinical trials has become necessary in order to understand how they interact and influence cardiovascular health [1–3]. However, some issues stem from the terminology biases among published literature: while the term “sex” should be used when reporting biological aspects, the term “gender” is more appropriate when reporting gender identity and psychosocial/cultural factors [1].

Ischemic heart disease (IHD) is the main cause of death among women and men [4]. Nonetheless, IHD has been perceived as a primarily “male disease”, and “evidence-based” clinical standards have been based on male pathophysiology and outcomes in basic and clinical research [5]. The low participation of women in clinical trials for CVD is cause of concern, especially in IHD patients [6, 7]. In fact, despite the decrease in CVD mortality over the past decade, trends are less favorable in women [8]. Several studies report clear sex discrepancies in clinical outcomes for IHD patients treated either with conservative or invasive approach (i.e., percutaneous coronary intervention (PCI)) [5, 7]. For example, in acute coronary syndrome (ACS), the benefits of early invasive strategy resulted less effective in women with high-risk profiles [9, 10], exhibiting a higher unadjusted in-hospital mortality in women undergoing PCI [9, 10]. So far, only two studies, the “Variation in Recovery: Role of Gender on Outcomes of Young AMI Patients” [11] and “GENdEr and Sex determInantS of cardiovascular disease: From bench to beyond-Premature Acute Coronary Syndrome” [12, 13], have explored the multilevel interactions between sex and gender in influencing CVD development and management in young patients with ACS [14].

### Beyond Epicardial Obstructive Coronary Artery Disease, Microvascular Dysfunction, and its Assessment

Microvascular perfusion impairment, not explained by an epicardial critical stenosis, is a common finding in IHD and it appears to provide an adjunctive prognostic value in IHD patients [15, 16]. Therefore, the term coronary microvascular dysfunction (CMD) was coined to provide an overarching definition that encompasses different clinical scenarios. The conundrum of CMD ranges from a reduced coronary flow reserve not explained by a flow-limiting epicardial stenosis to the coronary microvascular impairment coexisting with coronary artery disease (CAD). Specifically, CMD can be detected in IHD patients with obstructive CAD undergoing urgent (reperfusion CMD) or elective PCI (interventional CMD), as well as in cases of ischemia and no obstructive CAD (INOCA) [17]. CMD can only be indirectly assessed using invasive and noninvasive techniques that measure parameters strongly dependent on the functional and structural integrity of coronary microcirculation [18]. In the coronary catheterization laboratory, CMD is commonly diagnosed by evaluating coronary blood flow before and after administration of adenosine with a Doppler-tipped guide wire in the coronary artery [19]. However, the corrected thrombolysis in myocardial infarction (TIMI) frame count (cTFC) and myocardial blush grade (MBG) provide easy and accessible angiographic indexes of coronary blood flow that do not require additional coronary artery instrumentation [20]. The intra- and inter-observer reproducibility of cTFC and MBG is acceptable, and dye injection rate and catheter size do not affect its measurement [21, 22]. It is noteworthy that cTFC and MBG have been shown to correlate with other invasive and non-invasive measures of coronary blood flow [23–26]. The cTFC, used as a surrogate marker of coronary blood velocity, predicts coronary microvascular status [27]. Interestingly, abnormal cTFC and MBG have also been related to worse prognosis, although mostly in patients with ACS [28–30]. No association between cTFC and major adverse cardiovascular events (MACE) except angina hospitalization was reported in a pilot study of Women’s Ischemia Syndrome evaluation considering 298 patients with INOCA (defined as no stenosis ≥ 50% in any epicardial vessel), but the study population included only women [31]. Therefore, it remains unclear whether the prognostic implications of an abnormal cTFC or MBG vary in different categories of male and female patients with IHD-for example, in patients varying in clinical presentation, coronary anatomy, or type/extension of coronary lesions.

Preliminary data suggest that cardiac magnetic resonance (CMR) can identify a specific CMD pattern in IHD women [32], suggesting its potential role for recognizing sex-related differences in the conundrum of IHD [33]. This is raising interest in the potential use of CMR, especially to target women with atypical chest pain and to detect the extent of procedural CMD [18, 34, 35]. Over 100 women with CMD, diagnosed by invasive coronary reactivity testing, have showed reduced myocardial perfusion reserve index with vasodilator stress first-pass perfusion CMR compared with an age-matched reference group (*N* = 21). Moreover, the presence of diffuse myocardial fibrosis, documented by native myocardial T1 values assessed by CMR, has been reported in 14 women with CMD without obstructive CAD [32]. However, these data were collected in a female-only population and they lack the comparison with a matched male group.

### Mechanisms Responsible for Microvascular Dysfunction in Ischemic Heart Disease Patients: A Landscape to Explore Under the Lens of Sex

Microvascular impairment seen in diagnostic angiography and after PCI may be responsible for different degrees of myocardial injury and may represent an in vivo human model of ischemia–reperfusion myocardial damage [36]. The impairment of coronary flow owing to functional (i.e., impaired dilation or increased constriction of coronary microvessels) and/or structural abnormalities of the microcirculation can be sustained by several mechanisms [37, 38]. Recently, it has been proposed that CMD in women plays a central role in the genesis of symptoms and myocardial ischemia, specifically in the case of INOCA. Women more frequently experienced persistent ischemic symptoms, regardless of revascularization procedures, as compared to men. Therefore, CMD should be investigated in order to elucidate sex differences in clinical outcomes of IHD patients [39, 40].

Notably, a sex-specific response to myocardial ischemia has been demonstrated in pre-clinical studies [41]. It is unclear which factors contribute to sexual dimorphism in IHD; nevertheless, sex hormones with their multiple cardiovascular effects could play a crucial role [42], especially considering that the estrogen/androgen balance is involved in the cardioprotective response to ischemia–reperfusion injury [43].

In experimental models, females are at lower risk of ischemia–reperfusion damage and estradiol (E2) administered acutely to males can reduce the infarct size [44–46]. Indeed, the estrogen receptor (ER) beta versus ER-alpha receptor-mediated responses and the relative importance of genomic versus non-genomic effects are the subjects of considerable debate [43, 47]. The cardioprotective role of E2 in the modulation of ischemia–reperfusion injury has been extensively investigated: in models, E2 acts at multiple cellular levels during ischemia–reperfusion, including cardiomyocytes and fibroblasts [46, 48–51].

Despite plausible pathological pathways linking endogenous sex hormones and cardiovascular disease, prospective clinical studies have been contradictory. The imbalance between testosterone and E2 was looked at as a potential mechanism of ischemia and CAD development [52–55]. Recently, in postmenopausal women participating in the MESA (Multi-Ethnic Study of Atherosclerosis), a higher testosterone/E2 ratio was shown to be associated with the development of IHD and heart failure events, whereas higher levels of E2 are associated with a reduced risk of ischemic events [56]. Moreover, low levels of serum anti-inflammatory biomarkers are positively associated with sex hormones in male CAD patients [57] suggesting that the imbalance between sex hormones can influence the development of CAD.

### Platelets: Evidence Suggesting Sex Differences Sheds Light on the Potential Role of Hormone Balance

Sex hormones operate at the cellular level, including vascular cells and activated cells recruited at the site of the athero-thombotic process, primarily platelets [58]. Sex differences in platelet reactivity have been reported in response to agonists, regardless of any ongoing anti-platelet therapy [59].

Ex vivo evidence showed that platelets from women without CAD are more reactive than those of men in response to standard concentrations of agonist [60–62], with a greater expression (50–80% higher compared to men) of glycoprotein (GP) IIb/IIIa receptors. Among patients, platelets from women, and in particular Caucasian women, bound more fibrinogen in response to low and high concentrations of ADP and showed more spontaneous aggregation compared to men after adjustment for multiple cardiovascular risk factors [63].

Mechanisms responsible for differences in platelet function are largely unknown. Estrogen and androgen receptors are expressed in platelets [58]; therefore, platelets are an excellent model for studying the non-genomic effects of hormones [64]. Specifically, human platelets have been shown to express only ER-beta and androgen receptors (AR). The effects of E2 and of other forms of estrogen, as well as those of testosterone, have been only partially explored and with conflicting results. Ex vivo acute treatment of platelets with estrone or estriol was shown to increase aggregation in response to common agonists [65]. Conversely, 3-month replacement therapy with estrogen induced a decrease in agonist-induced platelet aggregation in treated patients compared to control groups [66]. Accordingly, chronic treatment with high levels of E2 in experimental models (i.e., mice) produced a significant reduction in platelet responsiveness both ex vivo and in vivo, with a simultaneous increase of bleeding time and resistance to thromboembolism [67].

Concerning the role of AR in the biology of platelet function, rat models have showed that the ex vivo aggregation of platelets isolated from males was greater than that exhibited by those isolated from females and correlates with higher levels of androgens [60]. Consistently, platelet aggregation is reduced following castration in male rats and restored by testosterone administration [68]. The acute treatment of both rat and human platelet-rich plasma with testosterone amplified the aggregation induced by common agonists, indicating its rapid non-genomic responses [69].

Platelets also play a key role in athero-thrombotic mechanisms. Therefore, exploring the effect of sex hormones on platelet function in IHD is a step forward toward a better characterization of biological differences in IHD between men and women.

### EVA Research Hypothesis and Aims

**Fig. 1:**
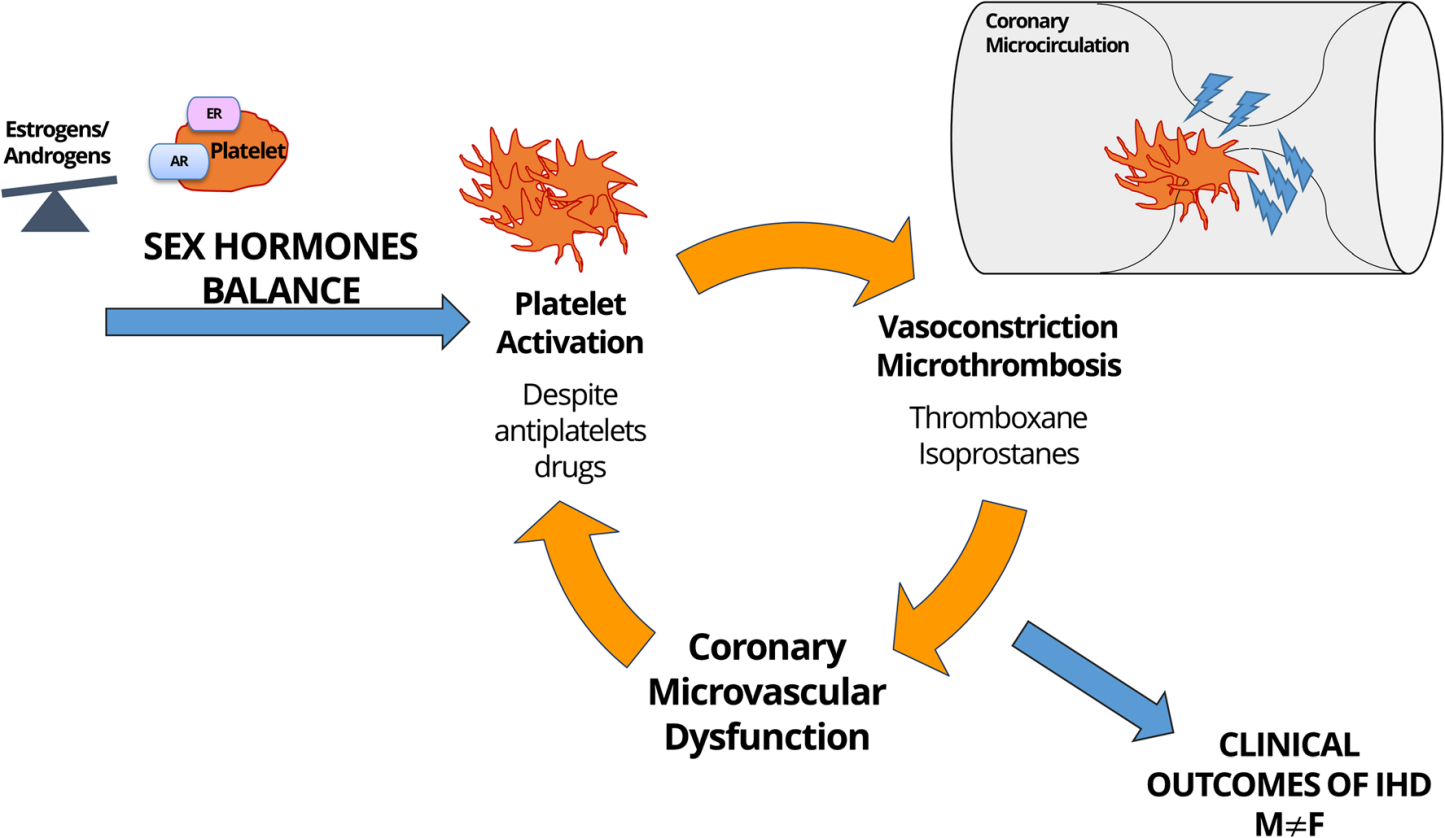
The EVA hypothesis: platelets, coronary microvascular dysfunction, and unbalanced clinical outcomes in women and men with ischemic heart disease

The research hypothesis is that women with IHD have a higher proportion of CMD due to a higher release of platelet-derived vasoconstrictor molecules such as thromboxanes, despite anti-platelet therapy. This status of hyper-activation might be modulated differentially by sex hormone balance among sexes according to age (Fig. 1). Further evidence on platelet biology and sex hormone interplay will provide mechanistic insights to understand CMD. Platelet activation markers will potentially be used as breakthrough innovative biomarkers to better predict outcomes of IHD patients.

We plan to conduct an observational study on patients affected by IHD undergoing angiography and/or PCI. Main objectives of EVA are:

1. To assess the sex–gender differences in severity of microvascular reperfusion damage in patients with IHD;
2. To evaluate estrogen/androgen effects on sex-related differences in myocardial ischemia–reperfusion damage occurring during PCI;
3. To investigate the differences in terms of platelet function between men and women affected by IHD and matched by age and clinical characteristics, according to CMD and CAD type;
4. To verify sex- and gender-driven interplay between the response to PCI procedure, platelet function, sex hormones, reperfusion imaging, and myocardial damage severity at baseline and its impact on outcomes at 12-month follow-up.

## Methods

### Study Design and Population

The “Endocrine Vascular disease Approach” (EVA) project (ClinicalTrials.gov identifier NCT02737982) was planned as an observational cohort prospective study. The flow chart of the study and the description of variables that will be considered are summarized in Table 1.

**Table 1:**
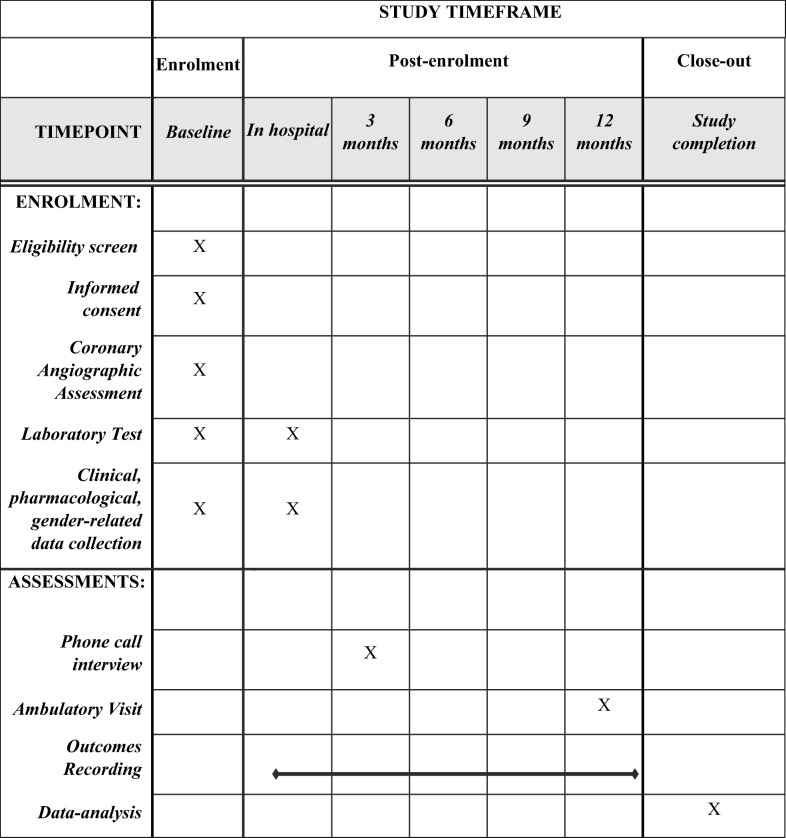
EVA Study design

A registry will be built up of men and women aged 18 and older with suspected IHD who were referred to the catheterization laboratory in order to undergo coronary angiography and/or PCI. Patients will be recruited according to the eligibility criteria reported in Table 2. Therefore, the EVA study population will include patients with stable CAD, patients with non-ST elevation (NSTE) ACS, and patients with ST elevation myocardial infarction (STEMI).

**Table 2:** EVA study population eligibility criteria

Inclusion criteria	Exclusion criteria
▪ Patients with ischemic heart disease (acute or chronic) undergoing percutaneous coronary intervention (urgent or elective) ▪ Written informed consent ▪ Both sexes ▪ Aged more than 18 years	▪ Life expectancy less than 12 months ▪ Active cancer (i.e., chemotherapy or ≤ 5 years from diagnosis) ▪ Pregnancy ▪ Previous coronary artery bypass graft ▪ Documented moderate–severe valvular heart disease ▪ Biological or mechanical valves

The study will be conducted in full conformance with the principles of the Declaration of Helsinki, the laws and regulations of Italy, or whichever affords the greater protection to the individual. The study has obtained the required authorization by the local Ethics Committee of Policlinico Umberto I, Sapienza University of Rome (reference 3786, 24/09/2015). Written informed consent will be obtained from all patients.

Among the study population, we will select (1) patients undergoing urgent PCI, specifically subjects who presented within 12 h of the onset of chest pain, who had ST segment elevation of more than 0.1 mV in two contiguous leads, and for whom the clinical decision was made to treat with PCI (STEMI); (2) patients with NSTE ACS defined by acute chest pain but no persistent ST segment elevation. ECG changes may include transient ST segment elevation, persistent or transient ST segment depression, T-wave inversion, flat T waves, or pseudo-normalization of T waves or the ECG may be normal. Myocardial ischemia can occur with (myocardial infarction–NSTEMI) or without cell loss (unstable angina); (3) patients undergoing coronary angiography and elective PCI for stable angina, with stress test inducible myocardial ischemia and de novo native coronary lesion with a stenosis of less 50% (no obstructive CAD) or > 50% stenosis in any epicardial artery (mono-vessel obstructive CAD). Patients will be treated according to standard guidelines based on the CAD type, either with PCI or coronary artery bypass graft.

The recruitment phase started in April 2016 and, currently, 200 patients have already been included in the study.

### Clinical, Pharmacological, and Gender-Related Factors

We will record complete information regarding (1) anthropometric data including height, weight, blood pressure, and heart rate; (2) socioeconomic status and gender-related factors: marital status, education level, income, physical activity (self-administered questionnaire), independent functional status Duke Activity Status Index [70], alcohol intake, smoking habits, perceived stress at work and at home, personality traits, and psychosocial characteristics and support [12, 71]; (3) cardiovascular risk factors (including familiar history of cardiovascular disease; hypertension; dyslipidemia, diabetes, previous cardiovascular events including previous MI and prior stent implantation); (4) symptom evaluation through standardized questionnaires [72, 73]; (5) dietary habits [74]; and (6) pharmacological therapy and Morisky Medication Adherence Scale—4 Items [75].

### Coronary Angiography Assessment

Coronary angiography will be performed according to standard methods. Qualitative and quantitative coronary angiographic analyses will be conducted by a core laboratory blinded to patient data. Time of procedure and timing to access at the catheterization after the first medical contact will be collected to explore chronobiology aspects.

#### Corrected TIMI Frame Count and Myocardial Blush Grade

The analysis of coronary flow will be done according to the TIMI frame count method by Gibson et al. [20, 28]. Values above the normal frame count will be considered suggestive of microcirculatory impairment. The mean TFC for each participant will be calculated using the cTFC of left anterior descending, left circumflex, and right coronary arteries. All participants with a TFC greater than two standard deviations from the normal published range in at least one of the three epicardial coronary arteries will be accepted as having micro-coronary slow flow, while those whose TFC falls within two standard deviations of the published normal range will be considered as having normal coronary flow.

The MBG [29] will be used to assess the filling and clearance of contrast in the myocardium. A rest or post-PCI MBG of less than 2 will define an impairment of coronary microcirculation [76].

#### Angiographic Definition of Extension and Localization of CAD

Based on angiography, we will classify IHD patients as follows: (1) ischemia with obstructive CAD, that is, ≥ 50% diameter stenosis; and (2) ischemia with no obstructive CAD (INOCA) < 50% diameter stenosis [17]. Based on the location of the CAD obstruction, we will identify one-vessel disease (1VD), two-vessel disease (2VD), and three-vessel disease or left main coronary artery disease (3VD) groups.

### Laboratory Measurements

Routine blood tests (e.g., cardiac troponin, as myocardial injury index, whole blood count, creatinine level, glycemia, lipid profile, hepatic enzymes) performed during hospitalization will be recorded.

The collection of biological samples for studies of platelet function will be performed in accordance with the guidelines suggested by the international literature and at scheduled time points. Blood samples will be properly maintained until batch analysis in freezers at − 80 °C. All samples will be coded to ensure anonymity and entered in conjunction with clinical data in a computerized database protected by double access code/password. All assays will be performed in a blinded fashion. The samples analyzed by immunoassay methods will be tested in duplicate, and those with concentrations exceeding the standard curve will be assayed again after appropriate dilution. The results obtained will be compared with reference values obtained in our laboratory on a reference population (healthy subjects, both sexes, and aged from 25 to 80 years).

During the angiography, blood samples will be collected at coronary and peripheral levels before and after PCI. According to a previously reported study, the arterial sample will be suitable for testing biomarkers of platelet function [77]. Moreover, a peripheral sample collection after 24 h will be performed.

Given the close interaction between thrombosis and hormone pathways, we plan to evaluate experimental biochemical markers of platelet function in association with sex hormone profiles.

In every patient, regardless of sex, we will perform the measurement of 17-beta estradiol (E2), luteinizing hormone (LH), follicle-stimulating hormone (FSH), and free testosterone (BioT). The BioT/E2 ratio will be calculated. For women, we will consider for the interpretation of results data concerning the menopausal status and, if in pre-menopause, the timing of the menstrual cycle in which the blood collection was performed.

To better understand sex differences, plasma, serum, and cell lysates opportunely prepared from the enrolled populations will be collected and managed in an appropriate manner. Furthermore, the platelet-rich plasma will be prepared for the study of platelet aggregation, and in vivo markers of platelet activation (i.e., thromboxane, soluble CD40 ligand, soluble P selectin, isoprostanes) will be detected. Apoptotic processes, protein expression of estrogens and androgens, and their signaling will all be measured in the cell lysates. Platelets will be used to study signal transduction pathways of sexual hormone receptors, nitric oxide synthase (NOS) phosphorylation, and oxidative stress pathways including NADPH oxidase 2 (NOX2). In these cell types, the protein expression of endocellular and membrane receptors will also be studied using Western blot. The involvement of the receptors will be investigated using specific inhibitors, hormones, and specific agonists.

Using flow cytometry, we will analyze platelet-derived microparticles and surface receptors (i.e., CD40 ligand, P-selectin, GPIIb/IIIa, CX3CR1, GPVI) from resting and activated platelets in vitro of patients with ACS versus age- and sex-matched healthy individuals. Therefore, we could evaluate the correlation between circulating molecules and membrane-associated markers.

### CMR Sub-Study

With the aim of better describing the CMR pattern of CMD in IHD patients, we planned a pilot study including (1) patients (*N* = 10; ratio W/M = 1:1) with mono-vessel obstructive CAD undergoing PCI and (2) patients (*N* = 10; ratio W/M = 1:1) undergoing angiography that documented no obstructive coronary disease with an impairment of microvascular dysfunction defined during angiography by a MBG < 2. Exclusion criteria will be (1) standard contraindication to magnetic resonance; (2) severe arrhythmia or tachycardia; (3) history or evidence of systemic autoimmune disorders, sarcoidosis, amyloidosis, or congenital heart disease; and (4) chronic kidney disease stages 4 and 5 (glomerular filtration rate < 30 mL/min/1.73 m^2^).

The procedure will be performed within 7 days from angiography to detect coronary microvascular dysfunction related to the procedure according to standard protocol (i.e., first-pass perfusion and the delayed post-contrast sequences). All CMR studies will be analyzed off-line in consensus by two experienced observers blinded to clinical information, using a workstation with dedicated cardiac software.

### Follow-Up Phase

The follow up-phase will include the in-hospital length of stay and 1 year after discharge. A phone call interview within 3 months and an ambulatory visit at 12 months will be performed.

Clinical, pharmacological, and behavioral information will be re-assessed at the time of follow-up. Information concerning the duration of in-hospital stay as well as any adverse events before discharge will be collected. The letter of discharge for each participant will be collected. Reassessment of medication adherence, as well as psychosocial and behavioral aspects will be checked at the scheduled follow-up as well as the occurrence of new comorbidities.

Adverse clinical outcomes will be determined through the medical chart reviews following the index angiography. Peri-procedural and after-discharge complications with focus on thrombotic and bleeding events will be recorded. Minor and major bleeding events according to International Society on Thrombosis and Hemostasis [78] and Bleeding Academic Research Consortium [79], as well as recurrence of angina symptoms will also be recorded during the follow-up phase.

All participants will be followed for 1 year to verify clinical endpoints: early (30 days) and late mortality (12 months) for cardiac-related events or for any other cause; recurrence of acute coronary syndrome; need for re-interventions or coronary artery bypass graft, complications related to PCI or re-admission for cardiac-related cause (i.e., heart failure), or for any other causes.

### Data Collection, Management, and Quality Assurance

The EVA coordination group (V.R., M.P., S.B., A.L.) will have full access to data and they will be responsible for the data management, including quality check. Investigators will be responsible for data entry into the electronic case report forms (eCRFs). In case of discrepant data, the coordination group will request data clarification, and if this should not be sufficient, the patient will be excluded from the analysis. EVA collaborators will receive training and a have access to a checklist for appropriate eCRF completion. By using a validation plan integrated in the data entry software, data will be checked for missing or contradictory entries and values out of the normal range. A final database will be created and validated by the study coordinator. Patients’ identification names will be registered by recruiting personnel. A series of consecutive numbers will identify patients. Regular monthly meetings will be conducted to check the sustainability of the project.

Source documents (paper or electronic) in which patient data are recorded will be collected. They will include, but not be limited to, hospital records, clinical and office charts, laboratory notes, patient-reported outcomes, evaluation checklists, pharmacy dispensing records, copies of transcriptions that are certified after verification as being accurate and complete, patient files with radiological images, and records kept at medico-technical departments involved in the trial. Source documents, required to verify the validity and completeness of data, must be retained per the policy for retention of records.

### Knowledge Translation

We will facilitate the dissemination of our research findings through publications (open access) and presentations at national and international congress meetings making scientific audiences aware of the findings and creating an interdisciplinary network that may influence both individual and public practice.

### Statistical Analysis Plan

The data will be analyzed by appropriate statistics. Student’s *t* test and Pearson correlation will be used for normally distributed variables. When necessary, log transformation will be used to normalize the data, or appropriate non-parametric tests will be employed (Mann–Whitney *U* test and Spearman rank correlation test). Adjusted multivariate analyses will be used to assess the differences and relationships between angiographic, laboratory, and clinical characteristics, stratifying the analysis on sex- and gender-related variables. Among the pre-specified subgroup analyses, we will explore the clinical presentation of ischemia (stable vs. acute coronary syndrome), the type of disease (obstructive vs. non-obstructive disease), traditional cardiovascular risk factors, medication adherence, and the gender construct including gender identity, gender role, gender relations, and institutionalized gender. We will consider the interaction term between sex and hormones in the changes of platelet biomarkers and angiographic index of coronary flow.

All calculations will be made using appropriate statistical software packages.

#### Sample Size Calculation

Preliminary data reported a higher rate of pathologic post-procedural MBG in women (50%) compared to men (30%) despite a TIMI flow grade 3 after PCI [80].

Therefore, the study requires 186 patients (93 per group, ratio men/women 1:1) to detect a difference of 20% in MBG < 2 at baseline and after PCI between men and women, with an alpha error equal to 5% and a power of 80%. Considering a drop-out rate of around 10%, total sample size required is 210 (105 per group).

### Clinical Significance and Translational Outlook of the EVA Project

The application of sex and gender medicine is strongly recommended by the World Health Organization and other international organizations [81–83]. The identification of biomarkers and therapeutic approaches that take sex and gender differences into proper consideration is crucial for the development of evidence-based medicine that reflects this important issue.

IHD is a complex and multifaceted syndrome and its pathophysiology includes different pieces of a single puzzle, including factors such as atherosclerosis, inflammation, platelet activation and vasospasm, as well as microcirculatory dysfunction worsened by abnormalities in endothelium, platelets, mitochondria, redox signaling, ion channels, and myocardium–blood flow cross-talk mismatch.

The EVA project will provide evidence regarding the contribution of platelet function and sex hormone balance in CMD. Moreover, the investigators expect to clarify the impact of biological sex and gender-sensitive variables on clinical outcomes after PCI procedure, including recurrence and re-hospitalization.

This study will inform on biological and psychosocial determinants of outcomes in IHD patients to be considered when developing a tailored approach aimed at improving the health of vulnerable patients. A better understanding of the interactions between sex hormones and platelet pathways involved in the development of ischemia will guide the development of potential pharmacological strategies based on sex, aiming, in turn, to modify the interaction between platelet and vascular responses in the setting of coronary angiography. Furthermore, the systematic collection of gender-related factors will contribute to the identification of the sex-specific impact of psycho-socio-cultural factors on clinical outcomes, providing new targets which will improve secondary prevention strategies and inform policies to foster the well-being of both women and men with IHD.

## Abbreviations

1VD: One-vessel disease
2VD: Two-vessel disease
3VD: Three-vessel disease
ACS: Acute coronary syndrome
AR: Androgen receptors
CAD: Coronary artery disease
CMD: Coronary microvascular dysfunction
CMR: Cardiac magnetic resonance
CVD: Cardiovascular disease
E2: Estradiol
eCRFs: Electronic case report forms
ER: Estrogen receptor
GP: Glycoprotein
IHD: Ischemic heart disease
INOCA: Ischemia and no obstructive CAD
MACE: Major adverse cardiovascular events
MBG: Myocardial blush grade
MMAS-4: Morisky Medication Adherence Scale—4 Items
NOS: Nitric oxide synthase
NOX2: NADPH oxidase 2
NSTEMI: Non-ST elevation myocardial infarction
PCI: Percutaneous coronary intervention
STEMI: ST elevation myocardial infarction
(c)TFC: (corrected) TIMI frame count
